# Optimization of Phenolic Compounds Extraction from Flax Shives and Their Effect on Human Fibroblasts

**DOI:** 10.1155/2017/3526392

**Published:** 2017-10-24

**Authors:** Magdalena Czemplik, Urszula Korzun-Chłopicka, Michał Szatkowski, Magdalena Działo, Jan Szopa, Anna Kulma

**Affiliations:** ^1^Department of Physico-Chemistry of Microorganisms, Institute of Genetics and Microbiology, Wroclaw University, Przybyszewskiego 63/77, 51-148 Wroclaw, Poland; ^2^Faculty of Biotechnology, Wroclaw University, Ul. Przybyszewskiego 63/77, 51-148 Wroclaw, Poland; ^3^Department of Genetics, Plant Breeding and Seed Production, Faculty of Life Sciences and Technology, Wroclaw University of Environmental and Plant Sciences, Wroclaw, Poland

## Abstract

The goal of this study was to evaluate the most effective technique for extraction of phenolics present in flax shives and to assess their effect on human fibroblasts. Flax shives are by-products of fibre separation, but they were found to be a rich source of phenolic compounds and thus might have application potential. It was found that the optimal procedure for extraction of phenolics was hydrolysis enhanced by the ultrasound with NaOH for 24 h at 65°C and subsequent extraction with ethyl acetate. The influence of the flax shives extract on fibroblast growth and viability was assessed using the MTT and SRB tests. Moreover, the influence of flax shives extract on the extracellular matrix remodelling process was verified. The 20% increase of the viability was observed upon flax shives extract treatment and the decrease of mRNA collagen genes, an increase of matrix metalloproteinase gene expression, and reduction in levels of interleukin 6, interleukin 10, and suppressor of cytokinin signaling 1 mRNA were observed. Alterations in MCP-1 mRNA levels were dependent on flax shives extract concentration. Thus, we suggested the possible application of flax shives extract in the wound healing process.

## 1. Introduction

Flax* (Linum usitatissimum)* is widely distributed in the Mediterranean region and temperate climate zone and plays an important role in the food industry and healthcare. The main beneficial properties of flax are associated with its oil and fibres. During fibre processing flax shives are separated and are usually considered waste material. The yield of shives is 2.5 tonnes for each tonne of fibre produced. Flax shives are a lignocellulosic fibre that helps the plant remain rigid during the growth phase and during production of seed. They are composed of 53% cellulose, 13% hemicellulose, 24% lignin, 1.5% of extractives, and 2% ash [1], although differences in the amounts of these polymers, proportions between them, and monomer compositions of flax fibre and shives are reported [2]. Flax shives are used as components in the furniture industry and in a range of forms including bulk transport trucks as well as paper and packaging products. Nevertheless, flax shives are a material conventionally considered a waste product of agricultural production.

For the purpose of this research, the previously described M50 genetically modified flax type characterized by production of polyhydroxybutyrate (PHB) in its fibres was used [3]. As a result of this modification, the M50 fibres exhibited improved mechanical properties, where PHB is bound to cellulose polymer by hydrogen and ester bonds during plant growth. M50 fibres were previously used in composite preparations [4], were applied as tissue engineering scaffolds [5], and were used for medical purposes as new dressings for chronic wounds with antibacterial properties [6]. The detailed analysis by GS–MS of the in vitro grown plants revealed that genetic modification resulted in altered phenylpropanoid levels [3]. Regarding the improved qualities of M50 flax fibres and their broad application possibilities, it is suggested that flax shives are also a source of bioactive compounds and thus might have application potential as well.

Phenylpropanoid compounds are normally found in most plant sources, including flax plants. They are a wide and important group of secondary metabolites, involved in plant growth, development, and plant defense against pathogens [7, 8]. They are also good antioxidants [9], possess antibacterial properties [10], and exhibit a wide range of therapeutic effects against various diseases including diabetes, cancer, and cardiovascular diseases [11]. Phenolic compounds, which are also present in flax shives, such as ferulic and* p*-coumaric acid and vanillin, are of special interest due to their biological activity favourable for human health [12–14]. Natural antioxidants are of particular interests in reducing the oxidative stress level in human organism. Some research reported that medicinal plants possess high antioxidant capacity and act at cellular level, through growth or proliferation stimulation, ROS scavenging, or lipid peroxidation [15–17]. Moreover, phenolic compounds, that is, phenolic acids, are known to regulate the normal human dermal fibroblast genes involved in antioxidant defense, the inflammatory response, and cell renewal [18]. Thus they are of great interests in aspect of wound healing or potential antiaging activity.

The main goal of the study was to optimize the extraction method of phenylpropanoids from flax shives and to evaluate their antioxidant potential and influence on normal human dermal fibroblasts cell line. First we established the optimal extraction and hydrolysis conditions regarding the putative influence of various parameters (solvent choice, time and temperature of hydrolysis, sodium hydroxide concentration, solvent type and volume, and numbers of extraction) on the extraction yield using the single-factor method. Then we determined the effect of ultrasonic treatment on the phenolic compounds yield. The phenolic contents of flax shives were studied in more detail in order to determine the impact of the modification on phenylpropanoid metabolism. In order to verify the putative biomedical application, mainly in wound healing of flax shives extract, we aimed to evaluate its effect on normal human dermal fibroblasts growth, proliferation, and influence on genes participating in extracellular matrix remodelling and inflammation.

## 2. Materials and Methods

### 2.1. Plant Material

Transgenic flax type M50 was generated and selected as described previously [3]. The flax (cv. Nike) plants were transformed using constructs bearing three of the genes necessary for polyhydroxybutyrate (PHB) synthesis (M plants). Constructs contained a plastidial targeting sequence. M50 plants were cultivated in a field and harvesting was carried out after 4.5 months. Retting using the dew method was conducted for twenty days. In this time the straw was turned over twice to ensure equal retting in the full straw volume. After drying, scutching and heckling the fibre was performed. During fibre processing, shives are obtained. M50 flax shives were used in this study.

### 2.2. Phenolic Extraction

Five grams of flax shives was ground using a Retsch mill and extracted with water or methanol or ethyl acetate thrice. Additionally, one sample was first hydrolysed at 65°C for 24 h, then the pH of the supernatants was adjusted to 3 and extraction with ethyl acetate was performed three times. The fractions were pooled and the solvents were dried under a vacuum. The remainder was resuspended in 1 mL of methanol and used for further analysis.

### 2.3. Determination of Total Phenolic Content

To determine the content of total free and ester bound phenolics the Folin–Ciocalteu method was used [19]. To an aliquot of the extract, diluted Folin–Ciocalteu reagent was added. Then, to each sample, saturated sodium carbonate and water were added. Total phenolic content was measured spectrophotometrically at 725 nm. The results are presented as gallic acid equivalents.

### 2.4. UPLC Analysis of Phenolics

The flax fabric extracts were analyzed using a BEH C18, 2.1 mm × 100 mm, 1.7 *μ*m column on a Waters Acquity UPLC system equipped with a 2996 PDA detector and quadrupole time-of-flight (QTOF) mass detector. The mobile phase was A = acetonitrile/B = 0.1% formic acid in a gradient flow: 1 min: 95% A and 5% B, 2–12 min: gradient to 70% A and 30% B, 12–15 min: gradient to 0% A and 100% B, and 15–17 min: gradient to 95% A and 5% B with a 0.4 mL/min flow rate. The mass spectra were acquired in ESI+ mode for 17 min in the range of 50–800 Da, under the following parameters: nitrogen flow 800 l/h, source temperature 70°C, desolvation temperature 400°C, capillary voltage 3.50 V, sampling cone 30 V, cone voltage ramp 40–60 V, and scan time 0.2 s. The components were identified on the basis of retention times, ultraviolet spectra, mass spectra, and comparison to authentic standards.

### 2.5. Alkaline Hydrolysis and Extraction of Phenolic Compounds: An Experimental Design

The effects of three variables (temperature: room temperature, 37°C, and 65°C; NaOH concentration: 2 M, 1 M, 0.5 M, and 0.2 M; and time 0–24 h) were investigated regarding their effect on NaOH hydrolysis yield. The fixed condition of the other variables was the following 3-time extraction with ethyl acetate using 100% solvent-to-solid material ratio. Five grams of flax shives was ground using a Retsch mill and hydrolysed at variable conditions. After hydrolysis, each sample was acidified to pH = 3 and underwent ethyl acetate extraction three times. The fractions were pooled and the organic solvent was dried under a vacuum. The remainder was resuspended in 1 mL of methanol and used for further analysis. Each treatment was performed in three replicates. The extraction yields in terms of the level of each identified phenolic compound (4-hydroxybenzoic acid, vanillic acid, vanillin, ferulic acid,* p*-coumaric acid, acetovanillone, and syringaldehyde) were determined with UPLC analysis.

### 2.6. Study for Determination of Appropriate Organic Solvent and Its Volume Used for Extraction

To determine the appropriate solvent range, ethyl, ethyl acetate: diethyl ether (1 : 1, v/v), and diethyl ether were used, as well as the number of extractions and solvent-to-solid material ratio (30%, 50%, and 100% v/m). The influence of each factor on the extraction yields was studied by the “one-factor-at-a-time” method. After hydrolysis at optimized conditions (2 M NaOH at 65°C for 24 h), each sample was acidified to pH = 3 and underwent extraction three times. The fractions were pooled and the organic solvent was dried under a vacuum. The remainder was resuspended in 1 mL of methanol and used for further analysis. Each sample was performed in three replicates. The extraction yields in terms of the level of each identified phenolic compound (4-hydroxybenzoic acid, vanillic acid, vanillin, ferulic acid,* p*-coumaric acid, acetovanillone, and syringaldehyde) were determined with UPLC analysis.

### 2.7. Ultrasonic Assisted Hydrolysis

An ultrasonic bath system (MKD-15 model, MKD Ultrasonic, Poland) was used for the purpose of this experiment. Ultrasonic energy was delivered with a relatively constant frequency of 40 kHz. The water temperature inside the bath was controlled and was 65°C. The ultrasonic treatment was performed for 15 min before, after, or before and after hydrolysis. After ultrasound-assisted hydrolysis, the extraction procedure of all samples was performed as described above, and each sample was acidified to pH = 3 and underwent extraction three times with 100% solvent-to-solid material ratio with ethyl acetate solvent.

### 2.8. Evaluation of Antiradical Activity

Radical scavenging activity of the flax shives extract was determined using the stable free-radical 2,2′-diphenylpicrylhydrazyl (DPPH) method. Aliquots of 6 *μ*l of the studied flax shives extracts were mixed with 200 *μ*l of 0.1 mM DPPH reagent (1,1-diphenyl-2-picrylhydrazyl). The samples were incubated at room temperature in darkness for 15 min, and then absorbance was measured at 515 nm. The control sample consisted of 200 mL of DPPH and 6 *μ*l of methanol. The optical densities of the resulting solutions were read at 517 nm using a Varioskan Flash plate reader (Thermo Scientific). The blank sample was pure methanol. The antioxidative properties were expressed as antioxidative potential (equal to the inhibition of the free-radical reaction expressed as a percentage) [20].

### 2.9. Cell Culture

Normal human dermal fibroblasts (NHDF, Lonza, USA) were maintained at 37°C, 5% CO2 in Minimum Essential Medium Alpha (*α*-MEM, Institute of Immunology and Experimental Therapy, Polish Academy of Science, Poland) supplemented with 10% fetal bovine serum (FBS, Lonza, USA), 1% L-glutamine (Invitrogen, USA), and 1% antibiotic mixture (Invitrogen, USA). NHDF used in this study were between the third and seventh passages.

### 2.10. Cell Growth and Proliferation Assay

NHDF cells were seeded at a concentration of 5 × 10^4^ cells/mL in a 24-well plate. The cell treatment with flax shives extracts (#1–#6) was performed. The extract was prepared from an equal amount (5 g) of dry weight of shives.  Table 4 represents the phenolic content in each preparation. The proliferation potential of NHDF cells was performed using MTT assay after 24 and 48 h of incubation. A 40 mL of MTT stock solution (4 mg/mL) was added to each well and after 4 h of incubation, the medium with MTT solution was removed from the plate. Then, 500 *μ*L of DMSO was added to each well to dissolve the formazan crystals. After 30 min of incubation with gentle shaking, the absorbance at 540 nm was measured on a Varioskan Flash Microplate Reader (Thermo Scientific, USA). The MTT assay was performed in four repetitions. The results were presented as a % in reference to the control (100%).

### 2.11. Cell Cytotoxicity Assay

To assess cytotoxicity against NHDF cells, the sulforhodamine B (SRB) assay was used as described previously (Skehan et al., 1990). After the cell treatment, as described above, the trichloroacetic acid (50 *μ*L/well, 50% w/v) was added for 1 h at 4°C in order to fix the cells, after 24 h and 48 h of incubation with flax shives extracts. Then, the plates were washed five times with distilled water and air-dried. The staining was performed with sulforhodamine B dye (0.4% w/v in 1% acetic acid, 40 *μ*L/well) and the unbound dye was washed 5 times with 1% acetic acid. After the plates were air-dried, the 10 mM Tris buffer (150 *μ*L/well, 10 mM) was used to dissolve the adsorbed dye, and the plates were gently shaken for 10 min on a mechanical shaker. The absorbance at 530 nm was read on a Varioskan Flash Microplate Reader (Thermo Scientific, USA). The cytotoxicity effect was calculated by subtracting the mean OD values of the respective blank from the mean OD value of the experimental set. The percentage growth in the presence of the test extract was calculated considering the growth in the absence of any test extracts as 100%.

### 2.12. Cell Culture Morphology Assessment

Cells were seeded in a 24-well plate on microscope cover slide at a concentration of 5 × 10^4^ cells/mL. After 24 h, flax shives extracts (#1–#6) were added to the plate. Flax shives extract was prepared from an equal amount (5 g) of dry weight of shives and the phenolic content in each preparation was presented in Supplementary Data, Table 1. To assess NHDF cells morphology, fibroblasts were analyzed in phase contrast microscopy and then stained with Hoechst (Sigma) for their nuclei analysis in a fluorescence microscope at 400x magnification.

### 2.13. RNA Purification and Real-Time PCR Analysis

The expression of genes involved in matrix remodelling, such as COLIA-1, collagen type 1, alpha 1; COLIA-2, collagen type 1, alpha 2; COLIIIA-1, collagen type 3, alpha 1; MMP-1, matrix metalloproteinase 1; MMP-2, matrix metalloproteinase 2; TIMP-1, metalloproteinase inhibitor 1; IL-6, interleukin 6; MCP-1, monocyte chemotactic protein 1 were determined by real-time PCR (RT-PCR). NHDF cells were seeded on 24-well plate at concentration of 20 × 10^5^ cells/mL. M50 flax shives extracts (shives #1, shives #2, and shives #3) were added to the plate. After 24 h, the cells were washed twice with PBS and then, the total RNA isolation was performed using the RNeasy Plus Kit (QIAGEN) following the manufacturer's protocol. The remaining DNA was removed via DNaseI (Invitrogen) treatment. Then, RNA was used as a template for cDNA synthesis using a High Capacity cDNA Reverse transcription Kit (Applied Biosystems). Real-time PCR reactions were carried out using a DyNAmoSYBR Green qPCR Kit (Thermo Scientific) on an Applied Biosystems Step One Plus Real-Time PCR System. Reaction conditions were designed according to the kit manufacturer's instructions. The oligonucleotide primer pairs for RT-PCR used in the present study were COLIA-1, 5′-GGGCAAGACAGTGATTGAATA-3′ (sense), 5′-ACGTCGAAGCCGAATTCCT-3′ (antisense); COLIA2, 5′-TCTCTACTGGCGAAACCTGTA-3′ (sense), 5′-TCCTAGCCAGACGTGTTTCTT-3′ (antisense); COLIIIA1, 5′-CGCTCTGCTTCATCCCACTAT-3′ (sense), 5′-CGGATCCTGAGTCACAGACAC-3′ (antisense); MMP1, 5′-GTTCCAAAATCCTGTCC-3′ (sense), 5′-CGTGTAGCGCATTCTGTCC-3′ (antisense); MMP2, 5′-AGATCTTCTTCTTCAAGGACCGGTT-3′ (sense), 5′-GGCTGGTCAGTGGCTTGGGGTA-3′ (antisense); TIMP1, 5′-CACCCACAGACGGCCTTATGCAAT-3′ (sense), 5′-AGTGTAGGTCTTGGTGAAGCC-3′ (antisense); MCP1, 5′-CCCCAGTCACCTGCTGTTAT-3′ (sense), 5′-T AGATCTCCTTGGCCACAATG-3′ (antisense); IL6, 5′-CCAGGAGCCCAGCTATGAAC-3′ (sense), 5′-CCCAGGGAGAAGGCAACTG-3′ (antisense); IL10, 5′-GAGGAGGTGATGCCCCAAGC-3′ (sense), 5′-TTCTTCACCTGCTCCACGGC-3′ (antisense); SOCS1, 5′-TTTTCGCCCTTAGCGTGAAG-3′ (sense), 5′-ATCCAGGTGAAAGCGGC-3′ (antisense). Reactions were carried out in three replicates. The GAPDH gene was used as a reference gene with the following primers: 5′-AGGTCGGAGTCAACGGAT-3′ (sense) 5′-TCCGGAAGATGGTGATG-3′ (antisense). The changes in transcript levels were presented as the relative quantification to the reference GAPDH gene, The GAPDH gene with the following primers: 5′-AGGTCGGAGTCAACGGAT-3′ (sense) 5′-TCCGGAAGATGGTGATG-3′ (antisense).

### 2.14. Statistical Analysis

Each test was performed in triplicate, and all data analysis was expressed as a mean ± standard deviation. ANOVA test was employed for statistical analyses of the results. The analyses were performed using Statistica 7 software (StatSoft, USA).

## 3. Results and Discussions

### 3.1. Effect of Solvent Type on Phenolic Compounds Content

Flax shives extracts were prepared using different solvents, such as methanol, water, and ethyl acetate. Additionally, alkaline hydrolysis with 2 M NaOH was performed with subsequent triple ethyl acetate extraction. It was expected to extract the phenylpropanoid compounds, and their total content was measured using the Folin–Ciocalteu method. The highest amount of phenolics was obtained for extraction with ethyl acetate with prior sodium hydroxide hydrolysis (7.14 mg/g), and the lowest amount was observed for extraction with ethyl acetate (0.74 mg/g). Similar results were obtained for the extraction method using water and methanol (3.93 and 4.29 mg/g, resp.) (Supplementary Data, Figure 1, in the Supplementary Material available online at https://doi.org/10.1155/2017/3526392). It is generally known that the yield of chemical extraction depends on the type of solvents used, but it was found that an important factor in this experiment was application of prior hydrolysis. It is well known that phenolic compounds exist in both free and bound forms in plant cells and that free phenolic compounds are solvent extractable, but bound phenolic compounds, which are covalently bound to the plant matrix, cannot be extracted into water or aqueous/organic solvent mixtures [21]. Therefore, prior hydrolysis with sodium hydroxide releases the phenolic compounds covalently bound with the cell wall polymers and thus contributes to the elevated level of total phenolics. Alkaline hydrolysis is also important for the stability of the phenolics in the extract [22].

Furthermore, we performed quantitative and qualitative analysis of phenolics in flax shives using ultra-performance liquid chromatography (UPLC) with a diode and mass detector. The comparison of the quantitative analysis of free phenolic compounds revealed the highest amounts for methanol extraction. Vanillin level (the most abundant compound in flax shives) reached 9.06 *μ*g/g in the water extract and 6.36 *μ*g/g in the methanol extract and was the lowest, 1.27 *μ*g/g, in ethyl acetate. The release of phenolic compounds by alkaline hydrolysis increased their level and was elevated 20-fold for vanillin (224.46 *μ*g/g). All identified metabolites are presented in Supplementary Data Table 1.

So far, mainly lignin, cellulose and hemicelluloses have been characterized in flax shives, but also other phenolic compounds are described [23, 24], and these have also been identified in the M50 flax type. Additionally, we demonstrated for the first time the presence of syringaldehyde and p-coumaric acid. We suggest that the introduced modification did not affect the composition of flax shives but slightly increased their level (Supplementary Data Table 1).

### 3.2. Optimization of Alkaline Hydrolysis Conditions

The previous experiments showed that alkali hydrolysis with sodium hydroxide results in a higher yield of phenolic compound extraction from flax shives. In order to optimize the alkaline hydrolysis conditions, the effects of temperature of hydrolysis, sodium hydroxide concentration, and duration of hydrolysis were examined.

**Figure 1:**
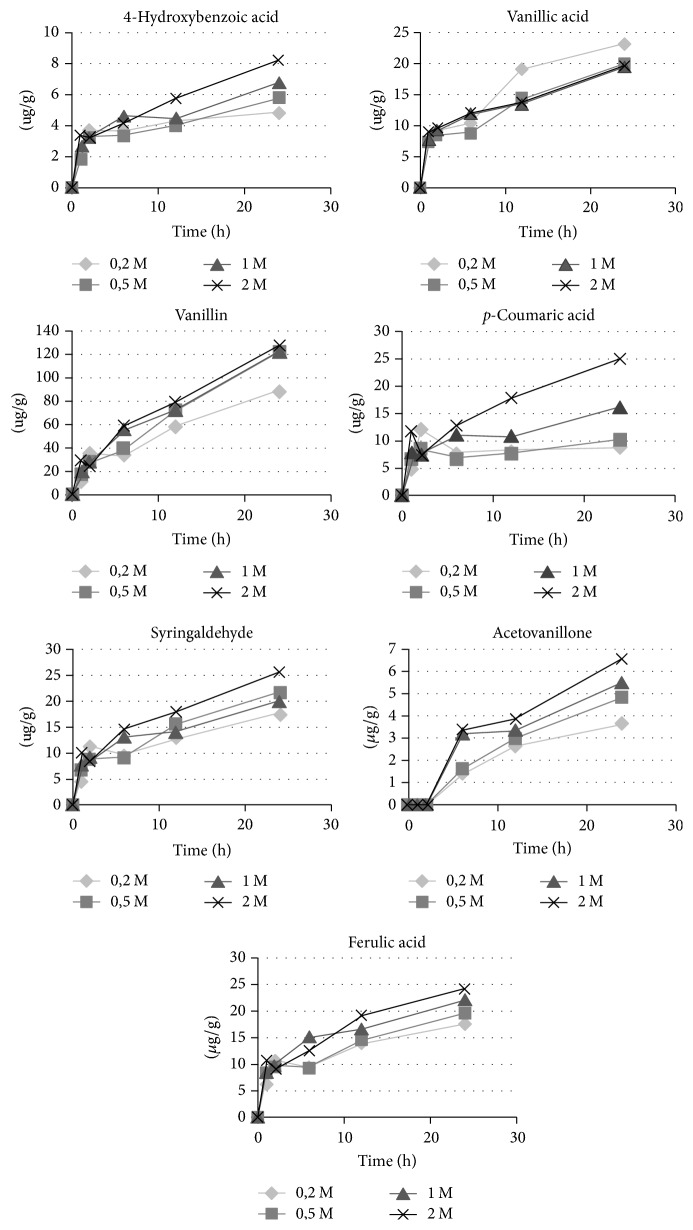
An influence of time of hydrolysis with sodium hydroxide and its concentration on phenolics content in flax shives extract. After the hydrolysis, each sample was acidified to pH = 3 and underwent three-time extraction with ethyl acetate using 100% solvent-to-solid material ratio.

Firstly, the effect of temperature on release of phenolic compounds from the cell wall was studied. The literature data concerning hydrolysis temperature differ; therefore room temperature, 37°C, and 65°C were tested. The UPLC quantitative analysis of identified phenylpropanoids is presented in Table 1. These results suggest that the extraction yields of phenolic compounds are very much temperature specific. The increase of temperature of hydrolysis to 65°C resulted in the nearly three times higher yield of phenylpropanoids (533.57 *μ*g/g) in comparison with room temperature (183.37 *μ*g/g), while the increase to 37°C elevated the total phenylpropanoids level to 270.12 *μ*g/g. Moreover, no phenolic compound disintegration was observed. It is thus suggested that the increase of temperature of alkali hydrolysis to 65°C increases the release of extracted compounds. Normally, increasing temperature promotes solubility of the compounds. However, plant phenolics are degraded or undergo enzymatic oxidation [25]. Therefore, higher temperature was not tested due to the possibility of disintegration of phenolic compounds and for economic reasons. Furthermore, we established the optimal time of hydrolysis with sodium hydroxide and its concentration. Four concentrations of NaOH were prepared, that is, 0.2 M; 0.5 M; 1 M; and 2 M, and used for flax shives hydrolysis. After 1, 2, 6, 12, and 24 hours, the samples were collected and underwent the standard procedure, which is acidification and ethyl acetate extraction. All samples were then analyzed with UPLC and the main components contents were determined. The results are presented in the graphs for each constituent separately (Figure 1).

Generally, in order to release higher concentrations of phenolic compounds, a higher concentration of sodium hydroxide is needed. All constituents of the flax shives extract exhibited the highest concentration after 2 M NaOH hydrolysis apart from vanillin, where above 0.5 M NaOH no differences were observed. For most identified compounds, the first hours of hydrolysis caused slow release of phenolic constituents covalently bound to the cell wall. After 12–24 h of hydrolysis, the amount of extracted compounds significantly increased, which was clearly seen in the example of vanillin or* p*-coumaric acid, where the concentration in 24 h of hydrolysis increased twofold in comparison to 12 h. It is worth noting that acetovanillone was not detectable in the first two hours of hydrolysis, and its content was determined after 6 h of hydrolysis. It was thus suggested that higher phenolic compounds concentration in flax shives extracts is reached after 24 h of hydrolysis with 2 M NaOH. Alternatively, if lower consumption of sodium hydroxide is necessary, 0.5 M NaOH may be used with a 10% lower extraction yield in comparison to the 2 M NaOH concentration. Similarly, prior alkali hydrolysis with 2 M NaOH has been reported to be an effective extraction method for flax lignan analysis in flax seeds [26]. It is also suggested that a long hydrolysis time is required to complete hydrolysis [27]. In the following experiments, 24 h hydrolysis with 2 M NaOH was applied.

### 3.3. Effect of Organic Solvent Used for Extraction

In this experiment, the effect of the organic solvent used for extraction after alkaline hydrolysis was assessed. Three-time extraction was performed using ethyl acetate, diethyl ether, or their mixture in a 1 : 1 ratio. The results clearly show that ethyl acetate is the most effective for phenylpropanoids extraction from flax shives (Table 2). The content of all identified phenylpropanoids was the highest for ethyl acetate extraction. Moreover, ethyl acetate exhibits lower vapour pressure than diethyl ether, and additionally it is superior for economic reasons. According to the available literature data, different solvents have been used for phenylpropanoids extraction from plant materials. Water, aqueous mixtures of ethanol, methanol, ethyl acetate, and acetone are commonly used to extract phenolic compounds from plants [28, 29]. Phenolic acids generally exist in a free, esterified, or glycosylated form in plants. Free and bound phenolic acids from rice were successively extracted using 70% ethanol at room temperature followed by centrifugation [30]. The highest amount of vanillin was found to be in the dehydrated ethanolic extract [31]. The highest levels of phenolics were extracted from* Vitis vinifera* wastes and sunflower meal using pure methanol and 80% aqueous acetone, respectively [32]. These differences could be due to the properties of the phenolic components of the plants concerned. Therefore, the choice of solvent of phenolic extraction compounds depends on their molecular structure. Less polar phenolic compounds can be extracted with hexane, and those with higher polarity can be extracted with methanol or ethanol. Furthermore, the minimal volume of ethyl acetate necessary for the extraction was verified as well as the number of extractions. The hydrolysed and acidified samples underwent extraction with 30%, 50%, and 100% of ethyl acetate compared to the extract volume. The results indicate that the 30% volume of the solvent is sufficient for effective extraction of phenolic from flax shives. The total amount of identified compounds was 131.61 *μ*g/g for 30% ethyl acetate (Table 3(a)) and 123.53 *μ*g/g for 100% (Table 3(c)). A higher yield was observed after using the 50% of volume of ethyl acetate, where the total amount of phenylpropanoids was 142.89 *μ*g/g (Table 3(b)). A statistically significant increase was observed in samples with use of 50% and 100% of ethyl acetate compared to the extract volume for the content of vanillin,* p*-coumaric acid, and syringaldehyde (Tables 3(b) and 3(c)).

Additionally, it was suggested that two extractions are sufficient, as in the case of the third one, only 3% of the total bioactive compounds are obtained. If the industrial scale is taken into account, it is therefore suggested that 30% ethyl acetate might be sufficient.

### 3.4. Enhancing Effect of Ultrasound on Extraction Yield

**Figure 2:**
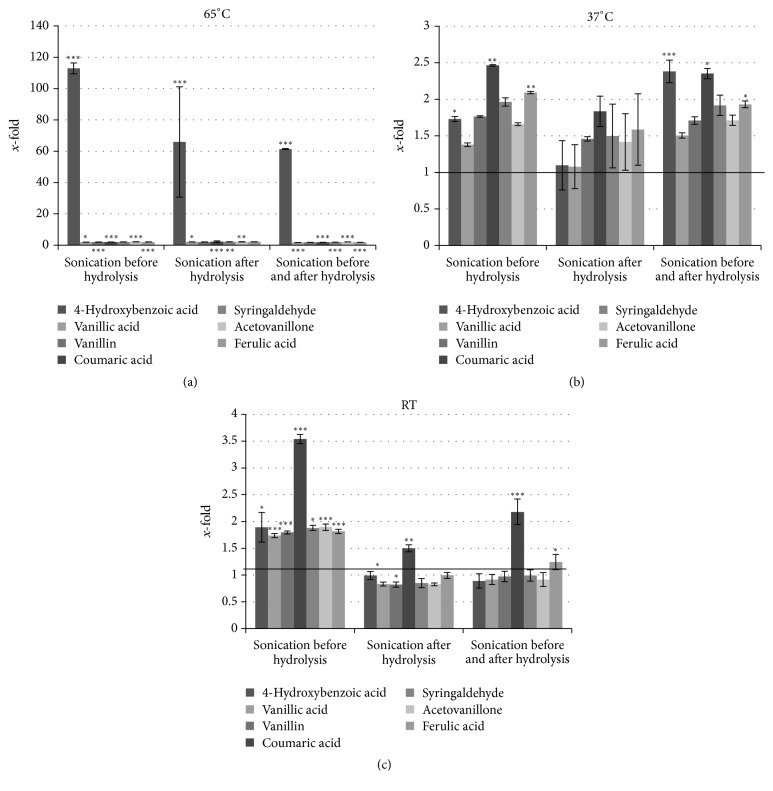
Effect of ultrasound on extraction yield of phenolic compounds in flax shives extract. The alkaline hydrolysis with 2 M NaOH at RT, 37°C, or 65°C for 24 h was performed; then, each sample was acidified to pH = 3 and underwent three-time extraction with ethyl acetate using 100% solvent-to-solid material ratio. For the determination of statistical significance ANOVA test was used (^*∗*^*P* < 0.05, ^*∗∗*^*P* < 0.01, and ^*∗∗∗*^*P* < 0.001).

As the previous experiment resulted in optimization of the method of phenylpropanoid extraction from flax shives, the effect of ultrasound treatment was verified. First, the effect of ultrasound treatment on hydrolysis at the optimal condition at 65°C was verified. Then, the temperature was lowered to 37°C and room temperature and ultrasound-assisted hydrolysis was performed. The results are presented in Figure 2. It was clearly shown that ultrasonic treatment enhanced the extraction yield. The content of each identified compound significantly increased when ultrasound was applied. However, no significant differences were seen in the phenolics yield between samples that were treated with ultrasound before, after, or before and after hydrolysis (Figure 2(a)). In all those samples, the content of each constituent increased twofold, except for 4-hydroxybenzoic acid, whose content increased 60-fold (sonication after hydrolysis and sonication before and after hydrolysis) and 100-fold (sonication before hydrolysis) (Figure 2(a)). It is suggested that one sonication is efficient in improving yield. Moreover, in order to lower the extraction costs, we aimed to verify whether the ultrasonic treatment could compensate the hydrolysis temperature influence with no negative effect on phenolic yield. Thus, we performed hydrolysis at room temperature and 37°C and applied ultrasound treatment. The results indicated that applying ultrasound at lower temperature of hydrolysis does not compensate the yield loss caused by lowering of temperature (Figures 2(b) and 2(c)). The twofold increase was only observed for samples sonicated before hydrolysis at RT (Figure 2(c)).

It is known that use of ultrasound induces acoustic cavitation which disrupts cell walls and facilitates the release of cell components. It increases solubility, diffusion, and penetration of the solvent into the plant cells, which significantly shortens extraction time and increases extraction yield. Additionally, polar molecules absorb ultrasonic radiation strongly because they have a permanent dipole moment [33]. It was shown that ultrasound can speed up the hydrolysis of conjugated phenolics in cranberry products and shorten the hydrolysis time [34]. Ultrasound-assisted extraction could improve the yield of total phenolics and total anthocyanins from wine lees [35]. Kawamura et al. showed the ultrasonic enhancement of the liquid carbon dioxide extraction of luteolin and apigenin from the leaves of* Perilla frutescens* [36]. Therefore ultrasound-assisted hydrolysis at 65°C is recommended for higher yield extraction of phenolics from flax shives.

### 3.5. Antioxidant Activity

**Figure 3:**
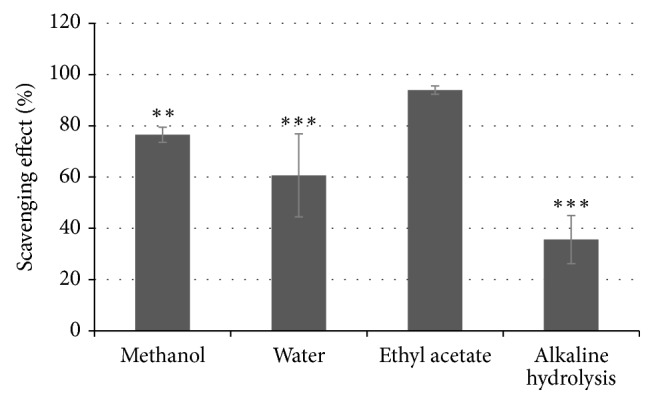
Antiradical potential of flax shives extracts determined with DPPH scavenging assay. The analyses were performed in three biological replicates. For the determination of statistical significance ANOVA test was used (^*∗∗*^*P* < 0.01 and ^*∗∗∗*^*P* < 0.001).

The antioxidant potential of tested flax shives extracts was determined using the DPPH method. The presence of phenolic compounds in the extracts influences the DPPH scavenging effect assay and depends on their ability to donate a hydrogen ion and convert the stable free radicals (DPPH^*∙*^) to nonradicals (DPPH-H). This change can be monitored spectrophotometrically by measuring the bleaching of DPPH^*∙*^ colour from violet to yellow at 515 nm. For the highest scavenging value, the strongest antioxidative properties are recorded. The antioxidant capacity was in accordance with the phenolics content in the extracts. The results show that % scavenging of DPPH^*∙*^ was higher in the extract obtained after alkaline hydrolysis and the subsequent ethyl acetate extraction and reached 35.6% (Figure 3). The worst scavenging effect was observed for ethyl acetate extract (93.9%). Methanol and water extracts exhibited percentage scavenging effect of DPPH^*∙*^ of 76.49% and 60.67%, respectively. The scavenging effect was in accordance with the phenolic compounds content in the extracts. Our findings support the theory that the radical scavenging effect is strictly related to the hydrogen atom donating ability of a compound. The ability of DPPH^*∙*^ scavenging increased with the increase of the total phenolic content. This is consistent with our previous result on the antioxidant activity of flax seedcake extracts. Antioxidant potential of extracts from seeds of the transgenic plants overproducing tannins or accumulating flavonoids was significantly increased compared to the control nonmodified extracts [37]. Phenolic compounds contribute to the overall antioxidant activities of the plant extracts, and these activities are closely related to their composition, as different compounds possess different antioxidative potential.

### 3.6. Influence of Flax Shives Extracts on Normal Human Dermal Fibroblasts

**Figure 4:**
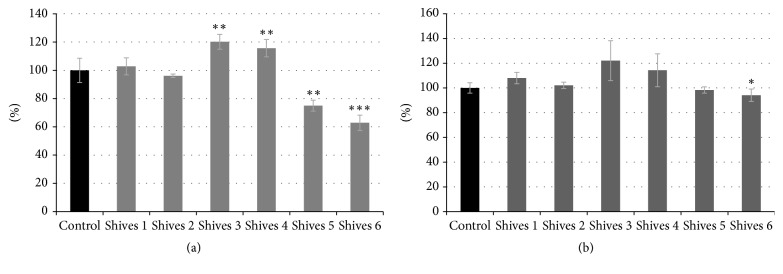
The effect flax shives extract treatment on NHDF cells in in vitro MTT test after 24 h (a) and 48 h (b). The numbers 1–6 correspond to the NHDF cells treated with shives extracts #1–#6. The analyses were performed in three biological replicates. The analyses were performed in three biological replicates. For the determination of statistical significance ANOVA test was used (^*∗*^*P* < 0.05, ^*∗∗*^*P* < 0.01, and ^*∗∗∗*^*P* < 0.001).

Due to the high phenolics content, flax shives extract might be putatively used in biomedical application. Therefore, their effect on human normal dermal fibroblasts cell line was evaluated. The effects of flax shives extracts #1–#6 on NHDF cells were assessed using the MTT test. Of the six preparations tested, #1–#4 exhibited no negative effect on NHDF viability. In those samples, viability was in range of 96.1% to 120.2% in comparison to the control, nontreated cells. An increase for preparation shives #3 and #4 reached 120.2% and 115.6%, respectively. Preparations #5 and #6 reduced a viability of NHDF cells, and a growth decrease of 24.9% and 37.1%, respectively, was observed after 24 h of treatment (Figure 4(a)). However, after 48 h of treatment no preparation exhibited any negative effect, and the viability of NHDF cells reached 94.1% to 122,1%. The highest viability was observed for shives #3 and #4 preparation and reached 122,1% and 114,3%, respectively (Figure 4(b)).

**Figure 5:**
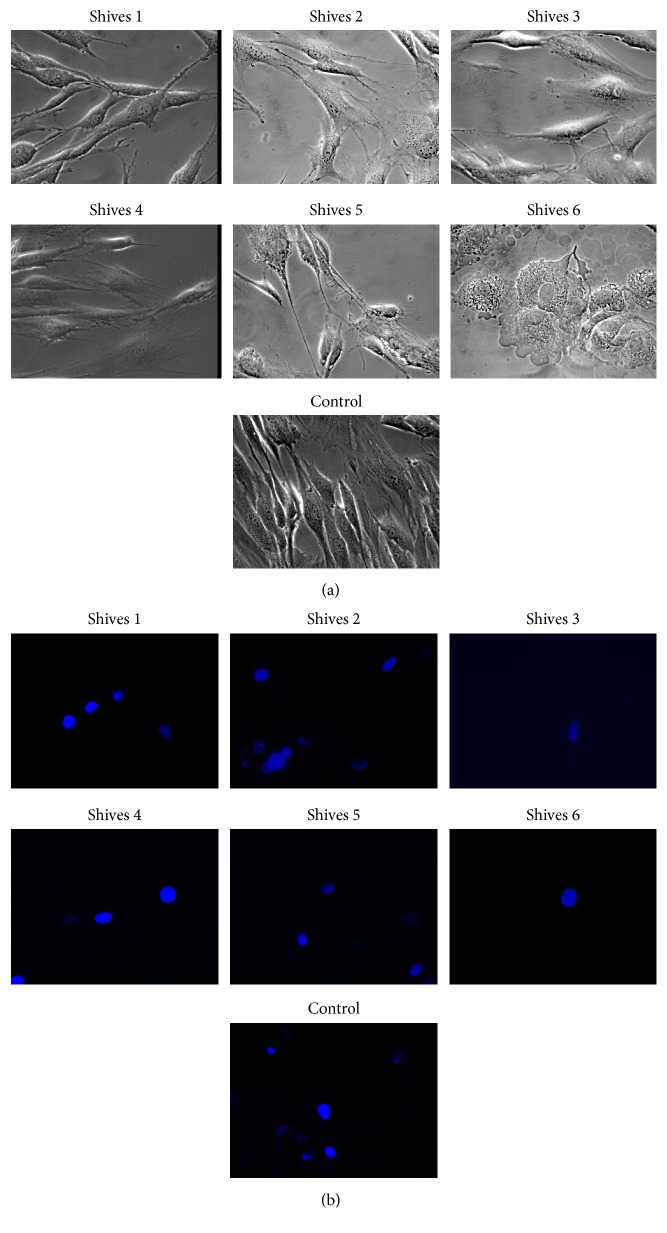
The effect of flax shives extract on morphology of NHDF cells. The fibroblast cells were analyzed under phase contrast microscope (a). The fibroblasts were also stained with Hoechst dye, and the nuclei of those cells were analyzed under a fluorescent microscope (b).

Moreover, flax shives extracts exhibited no cytotoxicity on human fibroblasts. The viable cells, as compared to untreated cells, comprised 92.4% to 103.5% after 24 h of incubation (Supplementary Data Figure 2(A)). Similarly, following treatment of fibroblasts with the flax shives extract for 48 h resulted in cell viability of 95.3%–105.7% (Supplementary Data Figure 2(B)). Furthermore the fibroblasts morphology and nuclei observations were performed. The NHDF cells treated with flax shives extracts #1–5# exhibited proper morphology, typical for fibroblast cells: flat and spread-out appearance, composed of regions of extensively spread cytoplasm. The NHDF cells treated with flax shives extract #6, with the highest concentrations of phenolics, did not exhibit the typical fibroblast morphology, and altered and impaired phenotype was observed. NHDF cells lost their original extended shape and were characterized with a rounded rather than elongated form (Figure 5(a)). Similar observations were performed after treatment of NHDF cells with flax seedcakes extracts, where cells were treated with extract with high concentration of phenylpropanoids were characterized with abnormal phenotype and inhibited growth [38]. This might be due to the fact that high concentration of phenolics is putatively toxic for fibroblasts. Furthermore, Hoechst staining revealed that the NHDF nuclei were similar in size and shape in all flax shives preparation tested. The majority of the cell nuclei had a near-circular shape (Figure 5(b)).

Flax shives extracted showed no negative effect on fibroblasts growth nor viability and thus proves to be nontoxic for human dermal fibroblasts. Moreover, the microscopic observation revealed the proper fibroblasts growth and phenotype. Our previous research revealed that fabric from M50 fibres exhibited the positive effect on cell proliferation, and no cytotoxicity against cultured fibroblasts was observed [6]. Fabric from transgenic M50 fibres contains i.a. 4-hydroxybenzoic acid, vanillic acid, vanillin, p-coumaric acid, syringaldehyde, and ferulic acid, similarly to M50 flax shives. Furthermore, research on flax seedcake preparations revealed that they increased NHDF cells proliferation by 30% [38] and their proliferative effect was due to the high content of phenylpropanoids. In our study, we observed the 20% increase in viability of fibroblasts; although it is not spectacular, these results might be a primary study for further analysis of the influence flax shives extract for putative skin application or for enhancers in wound healing processes. The granulation stage of wound healing process includes stimulation of fibroblast proliferation from the wound milieu and their migration into the wound during the epithelization stage [39].

### 3.7. Influence of Flax Shives Extract on mRNA Level of Genes Involved in Extracellular Matrix Remodelling in NHDF Cells

**Figure 6:**
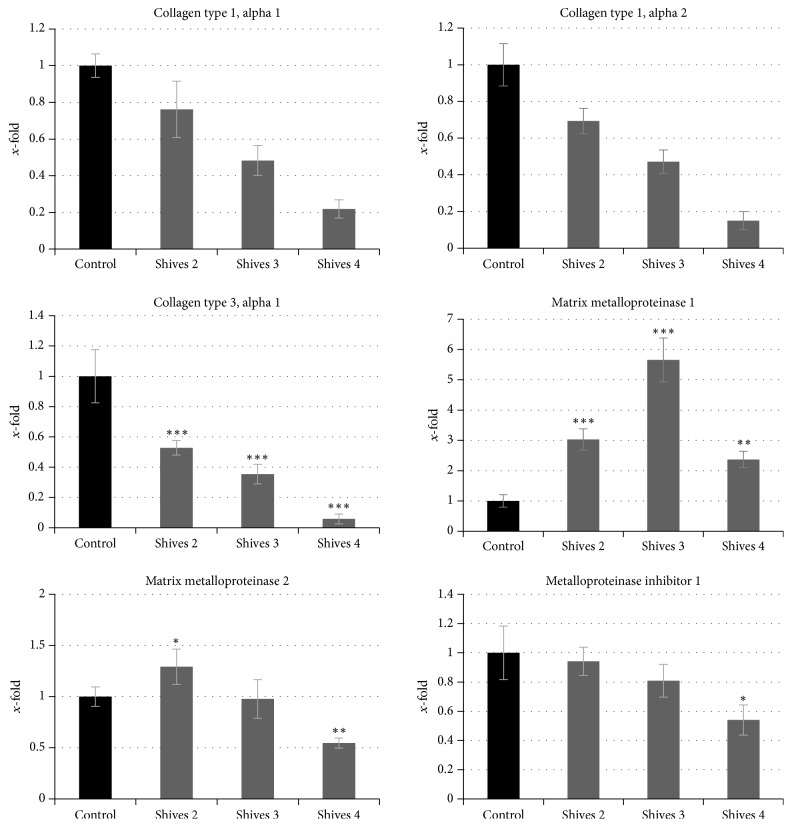
Influence of flax shives extracts on the level of expression of genes involved in extracellular matrix remodelling (collagen type 1, alpha 1; collagen type 1, alpha 2; collagen type 3, alpha 1; matrix metalloproteinase 1; matrix metalloproteinase 2, metalloproteinase inhibitor 1) in normal dermal human fibroblast. The analyses were performed in three biological replicates. For the determination of statistical significance Student's *t*-test was used (^*∗*^*P* < 0.05, ^*∗∗*^*P* < 0.01, and ^*∗∗∗*^*P* < 0.001).

In order to verify the influence of the flax shives extracts on the extracellular matrix remodelling in the human fibroblasts, the expressions of the genes coding for collagen type 1, alpha 1; collagen type 1, alpha 2; collagen type 3, alpha 1; matrix metalloproteinase 1; matrix metalloproteinase 2; and metalloproteinase inhibitor 1 were analyzed. On the basis of the previously performed experiments, three different shives extracts concentrations (#2, #3, and #4), which maintain viability of the NHDF cells the most, were used in the gene expression analysis. The obtained results are presented in Figure 6.

Generally, the expression level of the analyzed collagen genes in the fibroblasts treated with the shives extracts was significantly reduced in comparison to control cells. The most significant reduction of the collagen type 1 alpha 1, alpha 2, and collagen type 3 alpha 1 gene expression level was noted after shives #3 extract and was reduced by 78%, 85%, and 99%, respectively. On the contrary, the gene expression level of the matrix metalloproteinase remodelling genes were increased or barely changed in comparison to the untreated cells. The mRNA level of the matrix metalloproteinase 1 was significantly increased in the range between 2.4- and 5.6-fold, after incubation with shives extracts. Results for the matrix metalloproteinase 2 gene expression level were ambiguous. In comparison to the untreated NHDF cells, after treatment with shives extracts #2, #3, and #4, mRNA level of the metalloproteinase 2 was slightly increased (by 30%), unchanged, and reduced (by 55%), respectively. The gene expression level of the metalloproteinase inhibitor 1 was slightly reduced. Only for the shives extract #4, the significant decrease in the mRNA level of the metalloproteinase inhibitor 1 (by 46%) was noted. Regarding the above-presented results, it is suggested that extracts from M50 flax shives could find application in the wound healing process as they influence ECM rearrangement. Wound healing comprises several stages: hemostasis, inflammation, proliferation, and scar formation via rearrangements of the ECM. ECM rearrangement comprises degradation of fibrillar collagen I, collagen II, and collagen III by matrix metalloproteinase (MMP) [40]. The use of flax shives extract could therefore rearrange the ECM during last stages of wound healing process. Similar observations concerning the alteration in gene expression levels of genes involved in EMC remodelling were undertaken by Wojtasik et al. NHDF cells treatment with pectin isolated form flax shives exhibited significant influence on genes participating in extracellular matrix remodelling [41].

### 3.8. Influence of Flax Shives Extract on Inflammation-Related Genes in NHDF Cells

**Figure 7:**
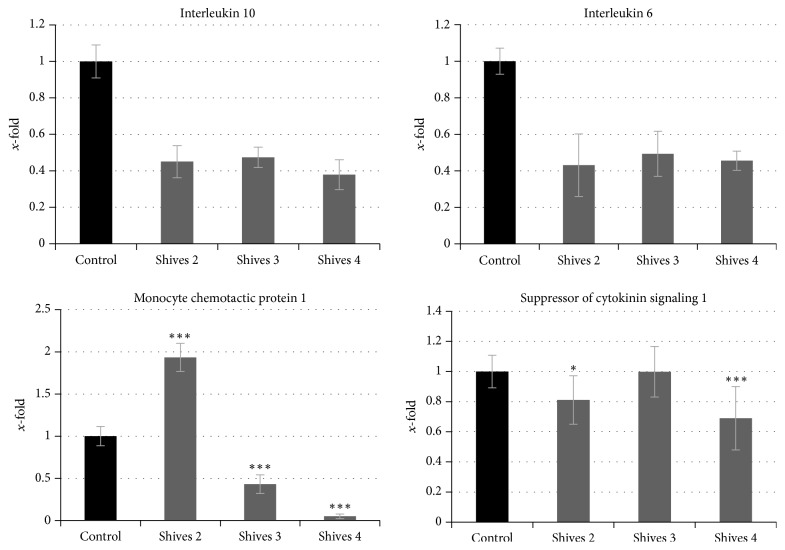
Influence of flax shives extract on the level of expression of genes' response to the inflammatory state (interleukin 6, interleukin 10, monocyte chemotactic protein 1, and suppressor of cytokinin signaling 1) in normal human dermal fibroblast. For the determination of statistical significance Student's *t*-test was used (^*∗*^*P* < 0.05 and  ^*∗∗∗*^*P* < 0.001).

In order to evaluate the impact of the flax shives extract on the inflammation process in the fibroblast cells, the expression level of genes coding for interleukin 6 (IL-6), interleukin 10 (IL-10), monocyte chemotactic protein 1 (MCP-1), and suppressor of cytokine signaling 1 (SOCS-1) was analyzed. The results are presented in Figure 7. In comparison to the untreated NHDF cells the flax shives extract significantly reduced the expression level of genes coding for analyzed interleukins. For IL-6 and IL-10, the decreased gene expression level in the range 43% to 49% and 38% to 47%, respectively, was noted. The expression level of the MCP-1 gene showed the significant modulation: increase after the shives #2 extract (by 93%) and decrease after the shives #3 extract (by 57%). However, more importantly, the shives #4 extract led to the massive reduction of MCP-1 gene expression level (0.1-fold). The analysis of the SOCS-1 gene revealed a moderate reduction in its expression level by shives #2 and #4 extracts (81% and 70%, resp.), whereas after the shives #3 extract any significant difference in comparison to the control was observed. The inflammatory response following tissue injury plays important role in wound healing [42] and restitution of equilibrium of pro- and anti-inflammatory processes is crucial for this process. The increase of MCP-1 expression results in the recruitment of mast cells that promote fibroblast proliferation during wound healing [43]. Moreover, MCP-1 can stimulate the macrophage response, which is crucial for this process [44]. Furthermore, there are some studies confirming that plant extracts can lower interleukin mRNA levels in order to reduce inflammation during the last stages of wound healing [45, 46]. SOCS1 is also involved in the wound healing process and is responsible for regulation of cytokine signaling during inflammation and macrophage activation [47, 48]. Treatment of NHDF with flax shives extract resulted in a reduction in SOCS-1 mRNA. Similar results were observed by Wojtasik et al. after fibroblast treatment with flax shives-derived pectin extract [41]. It is then suggested that flax shives extract might be effective in attenuating the inflammation state during last stages of wound healing process. We assume that flax-derived extracts possess high application potential for human use. Lately, we have demonstrated that flax straw metabolites effectively induced growth inhibition and apoptosis in human breast adenocarcinoma cells [49].

## 4. Conclusion

In summary, our results showed the most effective technique for extraction of phenolic compounds present in flax shives. Flax shives are by-products of fibre separation, but they are suggested to be a rich source of phenolic compounds and thus might have application potential. Therefore we decided to study the optimization method for their extraction. It was clearly shown that ultrasonic treatment enhanced the extraction yields of all compounds. Our investigation demonstrated that prior ultrasound-assisted hydrolysis with 2 M sodium hydroxide for 24 h at 65°C is necessary, with subsequent extraction with ethyl acetate. We qualitatively and quantitatively analyzed the phenylpropanoids of flax shives extract and identified the following main components: 4-hydroxybenzoic acid, vanillic acid, vanillin,* p*-coumaric acid, ferulic acid, syringaldehyde, and acetovanillone. The method has shown high sensitivity and selectivity, as well as good repeatability, and can be used as a standard method. Additionally, we suggest M50 flax shives extracts might be applicable in wound healing process, as they increased NHDF viability and exhibited no negative effect on fibroblasts growth and morphology and are not cytotoxic. Moreover, the influence of flax shives extract on genes coding for the extracellular matrix remodelling proteins revealed they decreased the mRNA level of collagen genes and increased the matrix metalloproteinase remodelling genes. Furthermore, the decrease in inflammation-related genes such as Il-6, IL-10, and SOCS1 was observed.

## Supplementary Material

Table 1: Identification and quantification of phenolic compounds in the M50 flax shives hydrolysed extract (A) and the vanillin content depending on the used extraction solvent (B).
Figure 1: Total phenolic content of flax shives extracts depending on the used extraction solvent. For the determination of statistical significance ANOVA test was used (∗∗ - P < 0.01, ∗∗∗ - P < 0.001).
Figure 2: The effect of flax shives extract on NHDF cells in in vitro SRB test after 24h (A) and 48h (B) of incubation. The numbers 1-6 correspond to the NHDF cells treated with shives extracts #1-#6. The analyses were performed in three biological replicates. For the determination of statistical significance For the determination of statistical significance ANOVA test was used.

## Figures and Tables

**Table 1:** Effects of temperature of alkaline hydrolysis on extraction yields of phenolic compounds in flax shives. The fixed conditions of the other variables were the following 3-time extraction with ethyl acetate using 100% solvent-to-solid material ratio. For the determination of statistical significance ANOVA test was used (^*∗*^*P* < 0.05 and ^*∗∗∗*^*P* < 0.001).

Compound	RT (*µ*g/g)	37°C (*µ*g/g)	65°C (*µ*g/g)
4-Hydroxybenzoic acid	7.01 ± 0.18	8.61^*∗*^ ± 0.62	15.81^*∗∗∗*^ ± 0.8
Vanillic acid	28.22 ± 0.72	36.48 ± 0.29	64.81^*∗∗∗*^ ± 8.6
Vanillin	102.07 ± 6.0	164.04 ± 2.3	347.04^*∗∗∗*^ ± 47.4
*p*-Coumaric acid	6.18 ± 1.18	8.47 ± 1.43	10.75^*∗*^ ± 2.07
Syringaldehyde	18.92 ± 0.6	26.32 ± 0.47	46.55^*∗*^ ± 5.04
Acetovanillone	4.09 ± 0.17	5.64 ± 0.31	18.98^*∗*^ ± 1.04
Ferulic acid	16.89 ± 1.27	20.56 ± 1.47	29.62^*∗*^ ± 2.81

Total	183.37	270.12	533.57

**Table 2:** Analysis of the solvent's effect on phenolics extraction yield in flax shives. Prior to the extraction the alkaline hydrolysis with 2 M NaOH at 65°C for 24 h was performed, and each sample was acidified to pH = 3 and underwent extraction with ethyl acetate, diethyl ether : ethyl acetate, 1 : 1, or diethyl ether. For the determination of statistical significance ANOVA test was used (^*∗*^*P* < 0.05, ^*∗∗*^*P* < 0.01, and ^*∗∗∗*^*P* < 0.001).

Compound (*µ*g/g)	Ethyl acetate	Diethyl ether : ethyl acetate, 1 : 1	Diethyl ether
4-Hydroxybenzoic acid	28.55 ± 1.1	23.42 ± 12	21.3 ± 0.06
Vanillic acid	53.1 ± 0.1	47.31 ± 0.5	47.43 ± 0.06
Vanillin	239.68^*∗*^ ± 7.0	216.17 ± 1.2	205.79 ± 0.4
*p*-Coumaric acid	60.91^*∗*^ ± 2.7	52.33 ± 2.0	48.43 ± 0.3
Syringaldehyde	26.18^*∗∗*^ ± 0.1	23.85^*∗∗*^ ± 0.3	24.23 ± 0.1
Acetovanillone	14.17 ± 0.5	13.04 ± 0.5	13.17 ± 0.0
Ferulic acid	71.6^*∗∗∗*^ ± 3.6	65.83^*∗∗*^ ± 0.7	64.56 ± 0.4

Total	494.18	441.95	424.9

**(a):** 

30%	Extraction I (*µ*g/g)	Extraction II (*µ*g/g)	Extraction III (*µ*g/g)
4-Hydroxybenzoic acid	4.8 ± 0.62	1.81 ± 0.15	0.8 ± 0.06
Vanillic acid	10.45 ± 1.46	3.13 ± 0.89	0.75 ± 0.17
Vanillin	66 ± 9.91	16.11 ± 3.31	2.8 ± 0.21
*p*-Coumaric acid	18.16 ± 2.14	3.84 ± 0.84	0.64 ± 0.21
Syringaldehyde	12.02 ± 1.34	3.2 ± 0.48	0.59 ± 0.06
Acetovanillone	3.39 ± 0.49	0.84 ± 0.16	0 ± 0
Ferulic acid	16.79 ± 2.15	3.71 ± 0.55	0.68 ± 0.13

Total	131.61	32.64	6.26

**(b):** 

50%	Extraction I (*µ*g/g)	Extraction II (*µ*g/g)	Extraction III (*µ*g/g)
4-Hydroxybenzoic acid	5.72 ± 1.0	1.74 ± 0.1	0.44 ± 0.06
Vanillic acid	11.2 ± 0.6	2.41 ± 0.3	0.45 ± 0.06
Vanillin	73.26^*∗∗∗*^ ± 5.5	12.91 ± 3	1.45 ± 0.5
*p*-Coumaric acid	18.58^*∗∗∗*^ ± 1.5	2.89 ± 0.9	0.3 ± 0.1
Syringaldehyde	13.16^*∗∗∗*^ ± 1.3	2.42 ± 0.6	0.31 ± 0.1
Acetovanillone	3.92 ± 0.4	0.55 ± 0.1	0.1 ± 0.03
Ferulic acid	17.06 ± 1.0	2.97 ± 0.4	0.26 ± 0.0

Total	142.89	25.89	3.31

**(c):** 

100%	Extraction I (*µ*g/g)	Extraction II (*µ*g/g)	Extraction III (*µ*g/g)
4-Hydroxybenzoic acid	5.41 ± 0.5	1.89 ± 0.2	0.25 ± 0.2
Vanillic acid	11.08 ± 0.9	3.07 ± 1.0	0.31 ± 0.2
Vanillin	62.99^*∗∗∗*^ ± 4.9	14.19 ± 4.4	1.1 ± 0.4
*p*-Coumaric acid	15.54^*∗∗∗*^ ± 1.3	3.93 ± 0.9	0.27 ± 0.1
Syringaldehyde	10.37^*∗∗∗*^ ± 0.9	2.99 ± 0.6	0.26 ± 0.0
Acetovanillone	3.24 ± 0.2	0.68 ± 0.3	0.1 ± 0.02
Ferulic acid	14.9 ± 1.2	3.9 ± 0.9	0.3 ± 0.1

Total	123.54	30.66	2.58

**Table 4:** Biochemical composition of M50 flax shives extracts used for cell in vitro studies.

Compound (*µ*g)	Extract number					
	*1*	*2*	*3*	*4*	*5*	*6*
4-Hydroxybenzoic acid	10.81	21.62	43.24	64.86	86.48	108.1
Vanillic acid	0.18	0.36	0.72	1.10	1.45	1.81
Vanillin	2.24	4.48	8.97	13.46	17.95	22.44
*p*-Coumaric acid	0.21	0.42	0.85	1.27	1.70	2.12
Syringaldehyde	0.13	0.26	0.53	0.80	1.06	1.33
Acetovanillone	0.09	0.19	0.38	0.58	0.77	0.96
Ferulic acid	0.38	0.77	1.54	2.31	3.08	3.85

## References

[1] Sain M., Fortier D. (2002). Flax shives refining, chemical modification and hydrophobisation for paper production. *Industrial Crops and Products*.

[2] J Y. (1992). Canadian nonwood pulp mill to start up in early. *Pulp Paper*.

[3] Wróbel M., Zebrowski J., Szopa J. (2004). Polyhydroxybutyrate synthesis in transgenic flax. *Journal of Biotechnology*.

[4] Szopa J., Wróbel-Kwiatkowska M., Kulma A. (2009). Chemical composition and molecular structure of fibers from transgenic flax producing polyhydroxybutyrate, and mechanical properties and platelet aggregation of composite materials containing these fibers. *Composites Science and Technology*.

[5] Gredes T., Kunert-Keil C., Dominiak M., Gedrange T., Wróbel-Kwiatkowska M., Szopa J. (2010). The influence of biocomposites containing genetically modified flax fibers on gene expression in rat skeletal muscle. *Biomedizinische Technik*.

[6] Kulma A., Skórkowska-Telichowska K., Kostyn K. (2015). New flax producing bioplastic fibers for medical purposes. *Industrial Crops and Products*.

[7] Cushnie T. T., Lamb A. J. (2006). Antimicrobial activity of flavonoids. *International Journal of Antimicrobial Agents*.

[8] Winkel-Shirley B. (2001). Flavonoid biosynthesis. A colorful model for genetics, biochemistry, cell biology, and biotechnology. *Plant Physiology*.

[9] Korkina L. G. (2007). Phenylpropanoids as naturally occurring antioxidants: from plant defense to human health. *Cell Mol Biol (Noisy-le-grand)*.

[10] Cowan M. M. (1999). Plant products as antimicrobial agents. *Clinical Microbiology Reviews*.

[11] Soobrattee M. A., Neergheen V. S., Luximon-Ramma A., Aruoma O. I., Bahorun T. (2005). Phenolics as potential antioxidant therapeutic agents: Mechanism and actions. *Mutation Research—Fundamental and Molecular Mechanisms of Mutagenesis*.

[12] Park S., Kim D. S., Kang S. (2011). Gastrodia elata Blume water extracts improve insulin resistance by decreasing body fat in diet-induced obese rats: vanillin and 4-hydroxybenzaldehyde are the bioactive candidates. *European Journal of Nutrition*.

[13] Srinivasan M., Sudheer A. R., Menon V. P. (2007). Ferulic acid: therapeutic potential through its antioxidant property. *Journal of Clinical Biochemistry and Nutrition*.

[14] Zang L.-Y., Cosma G., Gardner H., Shi X., Castranova V., Vallyathan V. (2000). Effect of antioxidant protection by p-coumaric acid on low-density lipoprotein cholesterol oxidation. *The American Journal of Physiology—Cell Physiology*.

[15] Singh D., Choi S. M., Zo S. M., Painuli R. M., Kwon S. W., Han S. S. (2014). Effect of extracts of *Terminalia chebula* on proliferation of keratinocytes and fibroblasts cells: an alternative approach for wound healing. *Evidence-Based Complementary and Alternative Medicine*.

[16] Hazra B., Sarkar R., Biswas S., Mandal N. (2010). Comparative study of the antioxidant and reactive oxygen species scavenging properties in the extracts of the fruits of *Terminalia chebula*, *Terminalia belerica* and *Emblica officinalis*. *BMC Complementary and Alternative Medicine*.

[17] Nakchat O., Meksuriyen D., Pongsamart S. (2014). Antioxidant and anti-lipid peroxidation activities of Tamarindus indica seed coat in human fibroblast cells. *Indian Journal of Experimental Biology*.

[18] Dudonné S., Poupard P., Coutiére P. (2011). Phenolic composition and antioxidant properties of poplar bud (*Populus nigra*) extract: individual antioxidant contribution of phenolics and transcriptional effect on skin aging. *Journal of Agricultural and Food Chemistry*.

[19] Gómez-Alonso S., Salvador M. D., Fregapane G. (2002). Phenolic compounds profile of Cornicabra virgin olive oil. *Journal of Agricultural and Food Chemistry*.

[20] Brand-Williams W., Cuvelier M. E., Berset C. (1995). Use of a free radical method to evaluate antioxidant activity. *LWT—Food Science and Technology*.

[21] Pérez-Jiménez J., Torres J. L. (2011). Analysis of nonextractable phenolic compounds in foods: The current state of the art. *Journal of Agricultural and Food Chemistry*.

[22] Vichapong J., Sookserm M., Srijesdaruk V., Swatsitang P., Srijaranai S. (2010). High performance liquid chromatographic analysis of phenolic compounds and their antioxidant activities in rice varieties. *LWT-Food Science and Technology*.

[23] Kim J.-W., Mazza G. (2006). Optimization of extraction of phenolic compounds from flax shives by pressurized low-polarity water. *Journal of Agricultural and Food Chemistry*.

[24] Kim J.-W., Mazza G. (2009). Extraction and separation of arbohydrates and phenolic compounds in flax shives with pH-controlled pressurized low polarity water. *Journal of Agricultural and Food Chemistry*.

[25] Davidov-Pardo G., Arozarena I., Marín-Arroyo M. R. (2011). Stability of polyphenolic extracts from grape seeds after thermal treatments. *European Food Research and Technology*.

[26] Cacace J. E., Mazza G. (2006). Pressurized low polarity water extraction of lignans from whole flaxseed. *Journal of Food Engineering*.

[27] Chen H., Zuo Y., Deng Y. (2001). Separation and determination of flavonoids and other phenolic compounds in cranberry juice by high-performance liquid chromatography. *Journal of Chromatography A*.

[28] Sun T., Ho C.-T. (2005). Antioxidant activities of buckwheat extracts. *Food Chemistry*.

[29] Amarowicz R., Estrella I., Hernández T. (2009). Antioxidant activity of a red lentil extract and its fractions. *International Journal of Molecular Sciences*.

[30] Harukaze A., Murata M., Homma S. (1999). Analyses of Free and Bound Phenolics in Rice. *Food Science and Technology Research*.

[31] Sharma A., Verma S. C., Saxena N., Chadda N., Singh N. P., Sinha A. K. (2006). Microwave- and ultrasound-assisted extraction of vanillin and its quantification by high-performance liquid chromatography in Vanilla planifolia. *Journal of Separation Science*.

[32] Casazza A. A., Aliakbarian B., Mantegna S., Cravotto G., Perego P. (2010). Extraction of phenolics from Vitis vinifera wastes using non-conventional techniques. *Journal of Food Engineering*.

[33] Tuulmets A., Raik P. (1999). Ultrasonic acceleration of ester hydrolyses. *Ultrasonics Sonochemistry*.

[34] Wang C., Zuo Y. (2011). Ultrasound-assisted hydrolysis and gas chromatography-mass spectrometric determination of phenolic compounds in cranberry products. *Food Chemistry*.

[35] Tao Y., Wu D., Zhang Q.-A., Sun D.-W. (2014). Ultrasound-assisted extraction of phenolics from wine lees: Modeling, optimization and stability of extracts during storage. *Ultrasonics Sonochemistry*.

[36] Kawamura H., Mishima K., Sharmin T. (2016). Ultrasonically enhanced extraction of luteolin and apigenin from the leaves of Perilla frutescens (L.) Britt. using liquid carbon dioxide and ethanol. *Ultrasonics Sonochemistry*.

[37] Zuk M., Prescha A., Stryczewska M., Szopa J. (2012). Engineering flax plants to increase their antioxidant capacity and improve oil composition and stability. *Journal of Agricultural and Food Chemistry*.

[38] Czemplik M., Kulma A., Bazela K., Szopa J. (2012). The biomedical potential of genetically modified flax seeds overexpressing the glucosyltransferase gene. *BMC Complementary and Alternative Medicine*.

[39] Skórkowska-Telichowska K., Czemplik M., Kulma A., Szopa J. (2013). The local treatment and available dressings designed for chronic wounds. *Journal of the American Academy of Dermatology*.

[40] Xue M., Jackson C. J. (2015). Extracellular matrix reorganization during wound healing and its impact on abnormal scarring. *Advances in Wound Care*.

[41] Wojtasik W., Czemplik M., Preisner M. (2017). Pectin from transgenic flax shives regulates extracellular matrix remodelling in human skin fibroblasts. *Process Biochemistry*.

[42] Koh T. J., DiPietro L. A. (2011). Inflammation and wound healing: the role of the macrophage. *Expert Reviews in Molecular Medicine*.

[43] Trautmann A., Toksoy A., Engelhardt E., Bröcker E.-B., Gillitzer R. (2000). Mast cell involvement in normal human skin wound healing: Expression of monocyte chemoattractant protein-I is correlated with recruitment of mast cells which synthesize interleukin-4 in vivo. *Journal of Pathology*.

[44] Wood S., Jayaraman V., Huelsmann E. J. (2014). Pro-inflammatory chemokine CCL2 (MCP-1) promotes healing in diabetic wounds by restoring the macrophage response. *PLoS ONE*.

[45] Paduch R., Wiater A., Locatelli M., Pleszczyńska M., Tomczyk M. (2015). Aqueous extracts of selected Potentilla species modulate biological activity of human normal colon cells. *Current Drug Targets*.

[46] Verma N., Amresh G., Sahu P. K., Mishra N., Rao C. V., Singh A. P. (2013). Wound healing potential of flowers extracts of Woodfordia fruticosa kurz. *Indian Journal of Biochemistry and Biophysics*.

[47] Feng Y., Sanders A. J., Ruge F., Morris C.-A., Harding K. G., Jiang W. G. (2016). Expression of the SOCS family in human chronic wound tissues: Potential implications for SOCS in chronic wound healing. *International Journal of Molecular Medicine*.

[48] Whyte C. S., Bishop E. T., Rückerl D. (2011). Suppressor of cytokine signaling (SOCS)1 is a key determinant of differential macrophage activation and function. *Journal of Leukocyte Biology*.

[49] Czemplik M., Mierziak J., Szopa J., Kulma A. (2016). Flavonoid C-glucosides derived from flax straw extracts reduce human breast cancer cell growth in vitro and induce apoptosis. *Frontiers in Pharmacology*.

