# Supplementary material for: Optimization of Phenolic Compounds Extraction from Flax Shives and Their Effect on Human Fibroblasts
Source: Evid Based Complement Alternat Med. 2017 Oct 24;2017:3526392. doi: 10.1155/2017/3526392 (PMC5674500; doi:10.1155/2017/3526392)
Supplement: Supplementary file 1 — Table 1: Identification and quantification of phenolic compounds in the M50 flax shives hydrolysed extract (A) and the vanillin content depending on the used extraction solvent (B). Figure 1: Total phenolic content of flax shives extracts depending on the used extraction solvent. For the determination of statistical significance ANOVA test was used (∗∗ - P < 0.01, ∗∗∗ - P < 0.001). Figure 2: The effect of flax shives extract on NHDF cells in in vitro SRB test after 24h (A) and 48h (B) of incubation. The numbers 1-6 correspond to the NHDF cells treated with shives extracts #1-#6. The analyses were performed in three biological replicates. For the determination of statistical significance For the determination of statistical significance ANOVA test was used. [file 3526392.f1.docx]

**Supplementary Data**

**Table 1** Identification and quantification of phenolic compounds in the M50 flax shives hydrolysed extract (A) and the vanillin content depending on the used extraction solvent (B).

**A**

| **Compound** | **µg/g** |
| --- | --- |
| 4-hydroxybenzoic acid | 21.62 ± 0,41 |
| vanillic acid | 18.14 ± 0.11 |
| vanillin | 224.46 ±.0,88 |
| ferulic acid | 21.28 ± 0.54 |
| *p*-coumaric acid | 13.37 ± 0.14 |
| acetovanillone | 9.68 ± 0.017 |
| syringaldehyde | 38.57 ± 0.37 |

**B**

|  | Vanillin (µg/g) |
| --- | --- |
| methanol | 6.36 ± 0.88 |
| water | 9.05 ± 2.83 |
| ethyl acetate | 1.26 ± 0.042 |
| hydrolysis with sodium hydroxide | 224.46 ±.0.88 |


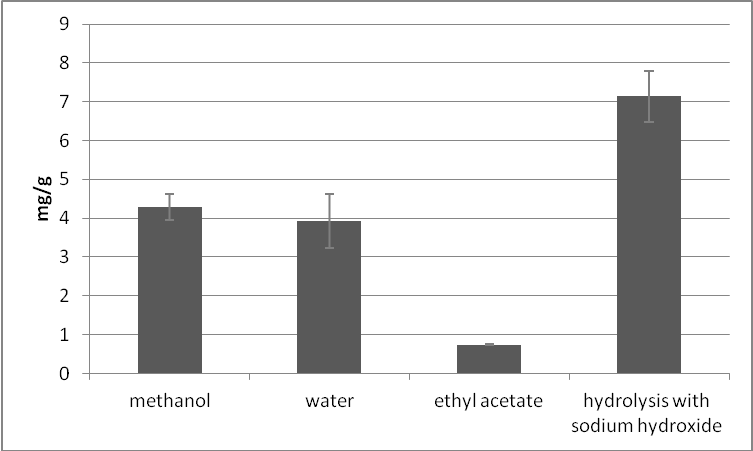


***

**

**

**Figure 1** Total phenolic content of flax shives extracts depending on the used extraction solvent. For the determination of statistical significance ANOVA test was used (** - P < 0.01, *** - P < 0.001).

(A)

(B)

**Figure 2** The effect of flax shives extract on NHDF cells in in vitro SRB test after 24h (A) and 48h (B) of incubation. The numbers 1-6 correspond to the NHDF cells treated with shives extracts #1-#6. The analyses were performed in three biological replicates. For the determination of statistical significance For the determination of statistical significance ANOVA test was used.
